# High-fat and high-sucrose diet impairs female reproduction by altering ovarian transcriptomic and metabolic signatures

**DOI:** 10.1186/s12967-024-04952-y

**Published:** 2024-02-12

**Authors:** Congcong Liu, Yunde Dou, Mengge Zhang, Shan Han, Shourui Hu, Yuxuan Li, Zhiheng Yu, Yue Liu, Xiaofan Liang, Zi-Jiang Chen, Han Zhao, Yuqing Zhang

**Affiliations:** 1 Institute of Women, Children and Reproductive Health, Shandong University, Jinan, 250012 Shandong China; 2 State Key Laboratory of Reproductive Medicine and Offspring Health, Shandong University, Jinan, 250012 Shandong China; 3 National Research Center for Assisted Reproductive Technology and Reproductive Genetics, Shandong University, Jinan, 250012 Shandong China; 4 Key Laboratory of Reproductive Endocrinology (Shandong University), Ministry of Education, Jinan, 250012 Shandong China; 5 Shandong Technology Innovation Center for Reproductive Health, Jinan, 250012 Shandong China; 6 Shandong Provincial Clinical Research Center for Reproductive Health, Jinan, 250012 Shandong China; 7 Shandong Key Laboratory of Reproductive Medicine, Shandong Provincial Hospital Affiliated to Shandong First Medical University, Jinan, 250012 Shandong China; 8 Research Unit of Gametogenesis and Health of ART-Offspring, Chinese Academy of Medical Sciences (No. 2021RU001), Jinan, 250012 Shandong China; 9 Department of Reproductive Medicine, Ren Ji Hospital, Shanghai Jiao Tong University School of Medicine, Shanghai, 200135 China; 10 Shanghai Key Laboratory for Assisted Reproduction and Reproductive Genetics, Shanghai, 200135 China

**Keywords:** Female reproduction, High-fat and high-sucrose, Polycystic ovary syndrome, Metabolism

## Abstract

**Background:**

Excessive energy intake in modern society has led to an epidemic surge in metabolic diseases, such as obesity and type 2 diabetes, posing profound threats to women’s reproductive health. However, the precise impact and underlying pathogenesis of energy excess on female reproduction remain unclear.

**Methods:**

We established an obese and hyperglycemic female mouse model induced by a high-fat and high-sucrose (HFHS) diet, then reproductive phenotypes of these mice were evaluated by examing sexual hormones, estrous cycles, and ovarian morphologies. Transcriptomic and precise metabolomic analyses of the ovaries were performed to compare the molecular and metabolic changes in HFHS mice. Finally, orthogonal partial least squares discriminant analysis was performed to compare the similarities of traits between HFHS mice and women with polycystic ovary syndrome (PCOS).

**Results:**

The HFHS mice displayed marked reproductive dysfunctions, including elevated serum testosterone and luteinizing hormone levels, irregular estrous cycles, and impaired folliculogenesis, mimicking the clinical manifestations of women with PCOS. Precise metabolomic overview suggested that HFHS diet disrupted amino acid metabolism in the ovaries of female mice. Additionally, transcriptional profiling revealed pronounced disturbances in ovarian steroid hormone biosynthesis and glucolipid metabolism in HFHS mice. Further multi-omics analyses unveiled prominent aberration in ovarian arginine biosynthesis pathway. Notably, comparisons between HFHS mice and a cohort of PCOS patients identified analogous reproductive and metabolic signatures.

**Conclusions:**

Our results provide direct in vivo evidence for the detrimental effects of overnutrition on female reproduction and offer insights into the metabolic underpinnings of PCOS.

**Supplementary Information:**

The online version contains supplementary material available at 10.1186/s12967-024-04952-y.

## Background

The unhealthy modern lifestyles, characterized by excessive calorie consumption and physical inactivity, have led to an epidemic surge in metabolic diseases, imposing a substantial burden on global public health [1, 2]. Overnutrition-induced disturbances in glucose and lipid homeostasis have triggered multiple metabolic disorders, such as obesity, type 2 diabetes (T2D), dyslipidemia, and non-alcoholic fatty liver disease. Of particular note, the prevalence of obesity is higher in women than in men [3]. According to World Health Organization estimates, up to 55% of women are overweight or obese, and obesity rates among women of reproductive age are still on the rise worldwide [4]. Apart from metabolic diseases, obesity also causes multisystem complications in females, including cardiovascular diseases, stroke, endometrial cancer, infertility, and polycystic ovarian syndrome (PCOS) [5].

Obesity stands as a primary risk factor that detrimentally affects women’s reproductive health. Obese women are more likely to exhibit lower ovarian reserve, irregular estrous cycles, and ovulation disorders [6, 7]. It has been reported that up to 88% of women with PCOS, the most common metabolic and reproductive disorder in women of reproductive age, are overweight or obese [8]. Furthermore, interventions aimed at weight reduction have demonstrated their efficacy in enhancing reproductive outcomes in women with PCOS [9, 10].

Another detrimental consequence driven by an energy surplus is the onset of T2D, characterized by hyperglycemia and insulin resistance, which could lead to female subfertility by impairing oocyte maturation, embryo quality, and endometrial receptivity [11, 12]. Hyperglycemia also represents a critical risk factor for adverse pregnancy outcomes, death, and disability among women globally [1]. Taken together, there exists an inseparable link between overnutrition and female reproductive health. Despite these clinical associations, the precise pathophysiology of how overnutrition affects female reproduction remains elusive.

In this study, we established a mouse model with obesity and hyperglycemia induced by a high-fat and high-sucrose diet, which mimics the modern lifestyle of overnutrition, to explore its impacts on female reproduction. Transcriptomic and metabolomic analyses were conducted to uncover ovarian molecular and metabolic alterations. Additionally, we compared this mouse model with a cohort of women diagnosed with PCOS and identified similar phenotypic signatures, suggesting a metabolic pathogenesis for this reproductive disorder in females.

## Methods

### Animal studies

Wild-type C57BL/6J mice were purchased from Gempharmatech (Jiangsu, China). To mimic the modern lifestyle of overnutrition, 8-week-old mice were fed a high-fat and high-sucrose (HFHS, 45% kcal fat, 35% kcal carbohydrate, Research Diets) or normal chow diet (NCD, 15 kcal% fat, Beijing KeaoXieli Feed) for 12 weeks. Each experiment was repeated three times with a sample size of a total of 4 to 15 mice per group. Mice were anesthetized with Avertin (250 mg/kg, i.p.) during tissue collection and euthanized by cervical dislocation after tissue removal. The mice were maintained on a 12-h light/dark cycle and had ad libitum access to food and water.

### Human studies

PCOS patients and control women were recruited from the Reproductive Hospital of Shandong University between January and June, 2014. The clinical and biochemical parameters were collected. PCOS patients (64 cases) of Han Chinese women were enrolled according to the Rotterdam criteria, requiring the presence of at least two of following criteria: oligo- or anovulation, clinical or biochemical signs of hyperandrogenism, and polycystic ovary with exclusion of some androgen-secreting diseases [13]. Another 68 unrelated Han Chinese women with normal menstrual cycles (26–35 days per cycle), hormone levels, and ovarian morphology were selected as the control group.

### Histology assessment

Mouse ovaries, subcutaneous white adipose tissue (SAT), visceral white adipose tissue (VAT, specifically the periovarian adipose tissue), and livers were isolated and fixed in 4% paraformaldehyde overnight at 4 °C. After dehydration and clearing, the tissues were embedded in paraffin and sectioned at 5 μm. Serial 5 µm ovarian sections were obtained and stained with hematoxylin and eosin (HE) for morphological observation and follicle counting. The thickness of theca cell layer was measured using ImageJ software (USA). We identified primordial follicles (characterized by a layer of flattened GCs), primary follicles (characterized by a single layer of cuboidal GCs), secondary follicles (characterized by more than one layer of cuboidal GCs without a visible antrum), and antral follicles (characterized by antral spaces) and quantified them as described previously [14]. We counted the follicles only when the oocyte nucleoli were visible.

### Hormone measurement

Mouse blood was collected through puncture of the retro-orbital plexus, centrifuged at 3000 rpm for 10 min for serum isolation. Serum was stored at − 80 °C until hormone measurement. Serum concentrations of testosterone (T), progesterone, and estradiol were measured using radioimmunoassay at the Beijing North Institute of Biological Technology (Beijing, China). The levels of luteinizing hormone and follicle stimulating hormone in serum were measured with ELISA kits (Elabscience) according to the manufacturer’s instructions.

### Estrous cycle assessment

To assess the estrous cycle of mice, vaginal swabs were collected daily for 13 consecutive days [15]. The vaginal epithelial cells were collected in 20 μL 0.9% saline, transferred to slides for air drying, and fixed with 95% ethanol. After hematoxylin and eosin staining, the estrous cycle was assessed according to the previously described criterion [16].

### Metabolic studies

Whole body lean mass and fat mass were measured in avertin-anesthetized mice using dual energy X-ray absorptiometry. Plasma total cholesterol (TC), low-density lipoprotein cholesterol (LDL-C), high-density lipoprotein cholesterol (HDL-C), and triglyceride (TG) levels were determined enzymatically according to the manufacturer’s guidance (Applygen). For the glucose tolerance test, mice were fasted overnight for 16 h and then injected intraperitoneally with 1.5 g/kg body weight glucose. For the insulin tolerance test, mice were fasted for 6 h starting at 8 am and then injected with 1 IU/kg body weight insulin. Blood glucose levels were measured with a portable glucometer (Accu-Chek, Roche) at 0, 15, 30, 60, and 120 min after injection. Serum insulin levels were measured by Mouse Ultrasensitive Insulin ELISA kits (Alpco) as described before [17].

### RNA isolation and qPCR analysis

Total RNA was extracted from ovaries using TRIzol reagent (Invitrogen) and reverse transcribed into cDNA using the Prime Script RT Kit with gDNA Eraser (Takara). The real-time PCR was performed using SYBR Premix Ex Taq Kit (Takara) on a Light Cycler 480 System (Roche). Results were normalized to the expression of housekeeping gene *Actin*. Primer sequences were listed in Additional file 1: Table S3.

### Ovarian precise metabolomics

The precise metabolomics, which is a robust non-targeted strategy for accurate quantitation and precise profiling of metabolomes [18], was conducted at LipidALL Technologies (Beijing, China). Polar metabolites were extracted from ovarian tissue using 500 µL of ice-cold methanol:H_2_O (4:1 ratio, vol/vol) containing 0.224 mM of Phenylhydrazine hydrochloride and two spoons of magnetic beads. Extracts were pooled into a single tube and dried in a SpeedVac under H_2_O mode. The dried extract was reconstituted in 2% acetonitrile in water prior to LC–MS analysis on an Agilent 1290 II UPLC coupled to Sciex 5600 + quadrupole-TOF MS. For reverse phase chromatography, polar metabolites were separated on a Waters ACQUITY HSS-T3 column (3.0 × 100 mm, 1.8 μm). MS parameters for detection were as follows: ESI source voltage positive ion mode 5.5 kV, negative ion mode − 4.5 kV; vaporizer temperature, 500 ℃; drying gas (N2) pressure, 50 psi; nebulizer gas (N2) pressure, 50 psi; curtain gas (N2) pressure, 35 psi; The scan range was m/z 60–800 [19]. Information-dependent acquisition mode was used for MS/MS analyses of the metabolites. Collision energy was set at 35 ± 15 eV. Data acquisition and processing were performed using Analyst^®^ TF 1.7.1 Software (AB Sciex, Concord, ON, Canada). PeakView 2.2 (AB Sciex, Concord, ON, Canada) was applied to extract MS/MS data and perform comparisons with the Metabolites database (AB Sciex, Concord, ON, Canada), HMDB and standard references to annotate ion identities [18]. A cocktail of 47 isotopically-labeled internal standards was spiked into the samples for metabolite quantitation. Peak areas of endogenous metabolites were normalized to the areas of their corresponding isotopically labeled structural analogues for quantitation.

### RNA-sequencing and analysis

Total RNAs of ovaries from mice subjected to either an NCD or an HFHS diet were extracted using TRIzol reagent. The extracted RNAs were used to construct RNA libraries with the NEBNext Ultra RNA Library Prep Kit for Illumina from New England Biolabs. These cDNA libraries were then sequenced using an Illumina Novaseq6000 platform by Gene Denovo Biotechnology (Guangzhou, China). Following sequencing, the obtained reads were subjected to a filtering process to remove any low-quality reads, and the remaining clean reads were aligned to a reference genome using the HISAT2 software (version 2.2.4). Bioinformatic analysis was conducted on OmicShare platform of Gene Denovo Biotechnology. Differentially expressed genes were identified with a *P*-value < 0.05 and a fold change > 1.5 between the two groups.

### Metabolomic and multi-omics data analyses

Principal component analysis (PCA), orthogonal partial least squares discriminant analysis (OPLS-DA), and corresponding score plot were conducted on the OmicShare platform. Pathway analysis overview, and metabolite set enrichment analysis were performed using MetaboAnalyst 5.0. To support data-driven network analysis, we have implemented the well-established debiased sparse partial correlation (DSPC) algorithm. Network graphs are optimized by Cytoscape. Using the joint-pathway analysis module of MetaboAnalyst 5.0, we performed integrated metabolic pathway analysis on results obtained from combined metabolomics and gene expression studies conducted under the same experimental conditions. Differentially metabolites were identified with the *P*-value < 0.05 and variable importance in projection (VIP) score > 1 between the two groups.

### Statistical analysis

The results are presented as mean ± SEM for the indicated number of observations. A two-tailed unpaired Student’s t-tests were conducted to compare two groups and one-way ANOVA for comparisons among multiple groups. Statistically significant differences were defined as *P* < 0.05.

## Results

### HFHS diet induces obesity and lipid metabolic disturbances in female mice

**Fig. 1:**
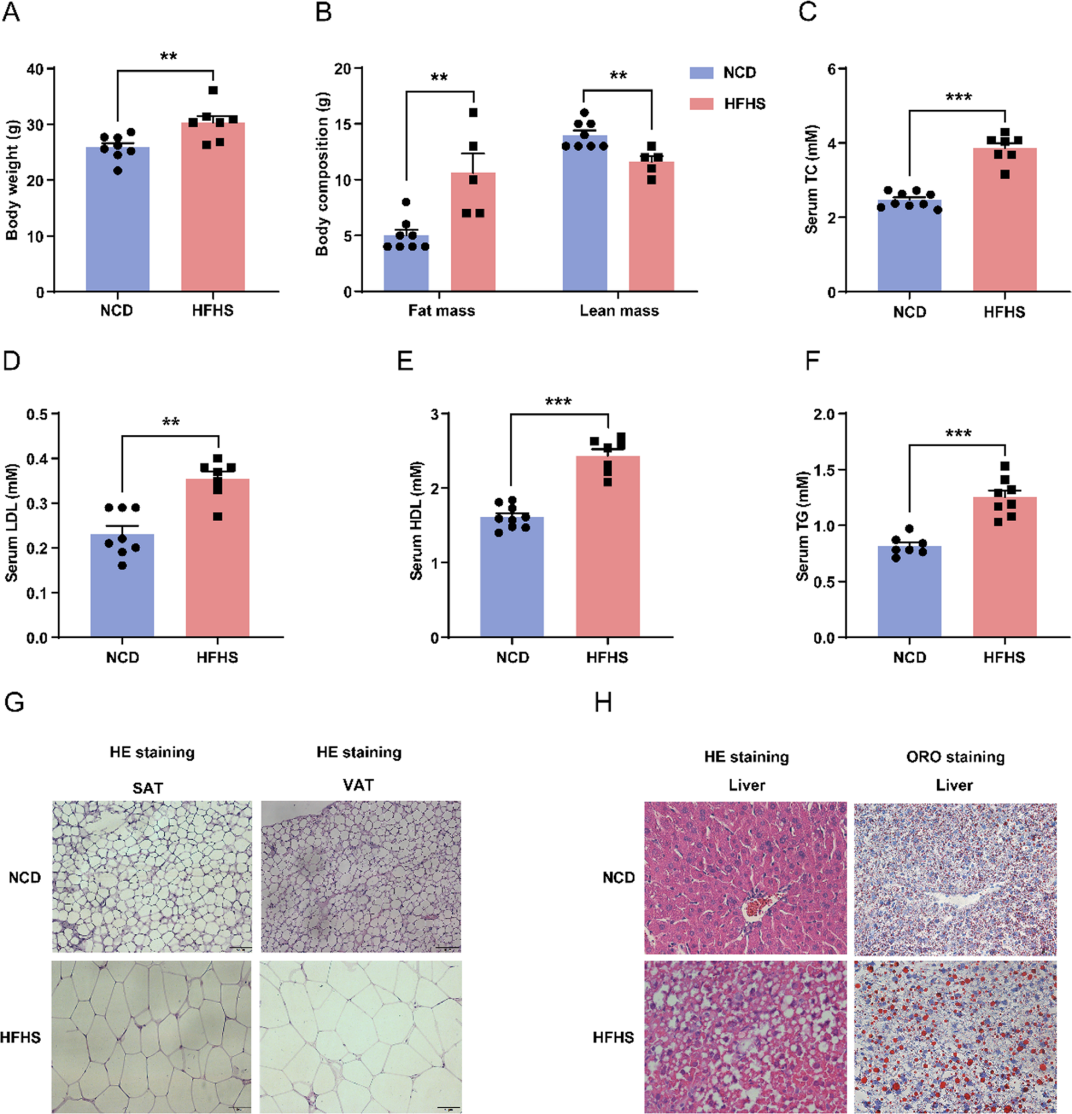
HFHS diet induces obesity and lipid metabolic disturbances in female mice. **A** Body weights of control and HFHS mice (control, n = 8; HFHS, n = 7). **B** Levels of fat mass and lean mass from control and HFHS mice detected by dual-energy X-ray absorptiometry (control, n = 8; HFHS, n = 5). **C** Serum levels of total cholesterol (TC) (control, n = 9; HFHS, n = 7). **D** Serum levels of low-density lipoprotein (LDL) (control, n = 8; HFHS, n = 7). **E** Serum levels of high-density lipoprotein (HDL) (control, n = 9; HFHS, n = 7). **F** Serum levels of triglyceride (TG) (control, n = 7; HFHS, n = 8). **G** Representative H&E staining images of SAT and VAT in control and HFHS mice. **H** Representative H&E and Oil Red O staining images of livers in control and HFHS mice. Data are presented as mean ± SEM, **P < 0.01, ***P < 0.001

To elucidate the impact of contemporary dietary patterns on female health, we established a high-fat and high-sucrose (HFHS) induced female mouse model. As expected, the body weight of these mice on the HFHS diet significantly increased compared to the normal chow diet (NCD) (Fig. 1A). Fat mass of the HFHS mice was increased by 2.12-fold compared to controls, while the lean mass was reduced by 17% (Fig. 1B), confirming the successful establishment of obesity. We further measured serum cholesterol and triglyceride levels in these mice. We found that the serum levels of total cholesterol (TC, Fig. 1C), low-density lipoprotein cholesterol (LDL-C, Fig. 1D), as well as high-density lipoprotein cholesterol (HDL-C, Fig. 1E) were all significantly elevated in HFHS mice. In addition, serum triglyceride (TG) levels were markedly increased by the HFHS diet (Fig. 1F). These results revealed that HFHS diet induces metabolic disturbances of both cholesterol and triglyceride in female mice.

Adipose tissue is a major site of lipid storage. In these HFHS mouse models, hematoxylin and eosin (H&E) staining revealed that subcutaneous white adipose tissue (SAT) adipocytes were significantly lager (Fig. 1G; Additional file 1: Fig. S1A). Studies have shown that SAT has a limited ability to increase its mass. When SAT reaches its maximum expansion capacity, lipids can spill over into liver and other intraperitoneal tissues [20]. We then examined the morphology of visceral white adipose tissue (VAT), specifically the periovarian adipose tissue. We observed that the sizes of VAT adipocytes in HFHS mice were enlarged, and quantification analysis showed that their mean area was 23-fold larger than that of the control group (Fig. 1G; Additional file 1: Fig. S1B). Since adipocyte size reflects the balance between triglyceride storage and mobilization, and the liver is the central regulatory organ for lipid metabolism. To investigate whether HFHS diet disrupts hepatic lipid metabolism, we performed H&E and Oil Red O (ORO) staining and identified striking steatosis and lipid accumulation in hepatocytes of HFHS mice (Fig. 1H). Taken together, these findings demonstrate that the HFHS diet causes obesity, hypercholesterolemia, hyperlipidemia, and non-alcoholic fatty liver disease in female mice.

### HFHS diet leads to hyperglycemia and insulin resistance in female mice

**Fig. 2:**
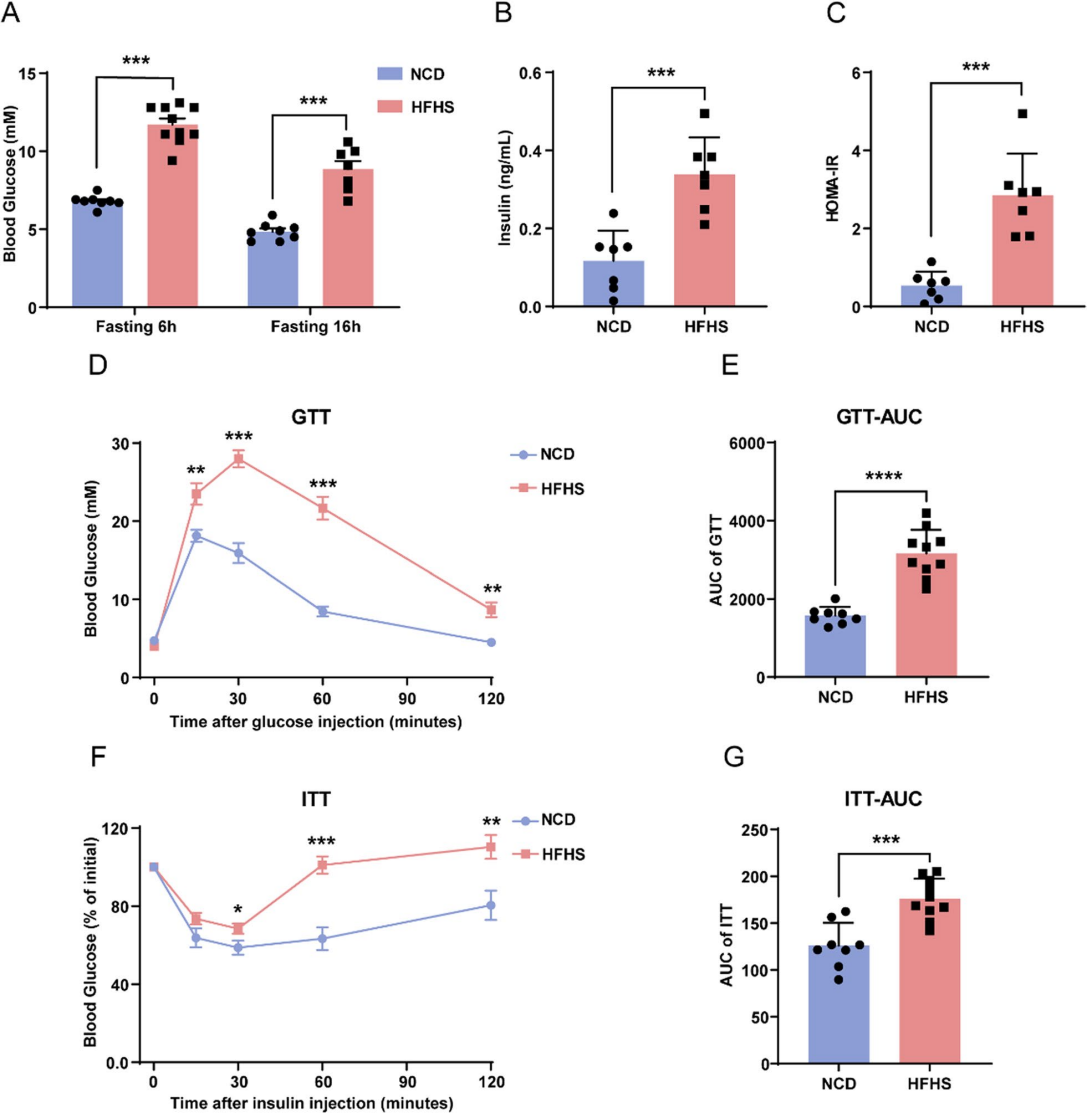
HFHS diet leads to hyperglycemia and insulin resistance in female mice. **A** Blood glucose levels in control and HFHS mice after fasting for 6 h or 16 h (control, n = 8; HFHS, n = 7–10). **B** Serum insulin levels of control and HFHS mice after fasting for 6 h (n = 7). **C** HOMA-IR in control and HFHS mice after fasting for 6 h (n = 7). **D**, **E** GTT and the related AUC in control and HFHS mice (control, n = 8; HFHS, n = 10). **F**, **G** ITT and the related AUC in control and HFHS mice (control, n = 8; HFHS, n = 10). Data are presented as mean ± SEM, *P < 0.05, **P < 0.01, ***P < 0.001, ****P < 0.0001

To further determine the effect of HFHS diet on glucose homeostasis, we analyzed fasting blood glucose (FBG) levels of these mice. The 6-h and 16-h FBG levels were significantly elevated after HFHS feeding, reaching 11.7 and 8.9 mM, respectively (Fig. 2A). Correspondingly, their serum insulin levels increased by 65% after HFHS feeding (Fig. 2B). HOMA-IR, an index that indicates the degree of insulin resistance, also significantly increased in the mice subjected to HFHS diet (Fig. 2C).

Using the glucose tolerance test (GTT), we discovered a significant impairment of glucose tolerance in HFHS mice, with a 2-h blood glucose level of 8.7 mM in the HFHS group, 1.93-fold compared to controls (Fig. 2D). The area under the curve (AUC) values were approximately twice those of the control group (Fig. 2E). We further directly examined the insulin sensitivity of these mice. The insulin tolerance test (ITT) revealed elevated blood glucose levels after insulin administration in HFHS mice, highlighting a suppressed capacity for insulin-induced glucose clearance (Fig. 2F, G). Therefore, HFHS diet causes hyperglycemia, impaired glucose tolerance, and insulin resistance in female mice, reflecting diabetic phenotypes.

### HFHS diet causes hormonal imbalance and irregular estrous cycles in mice

**Fig. 3:**
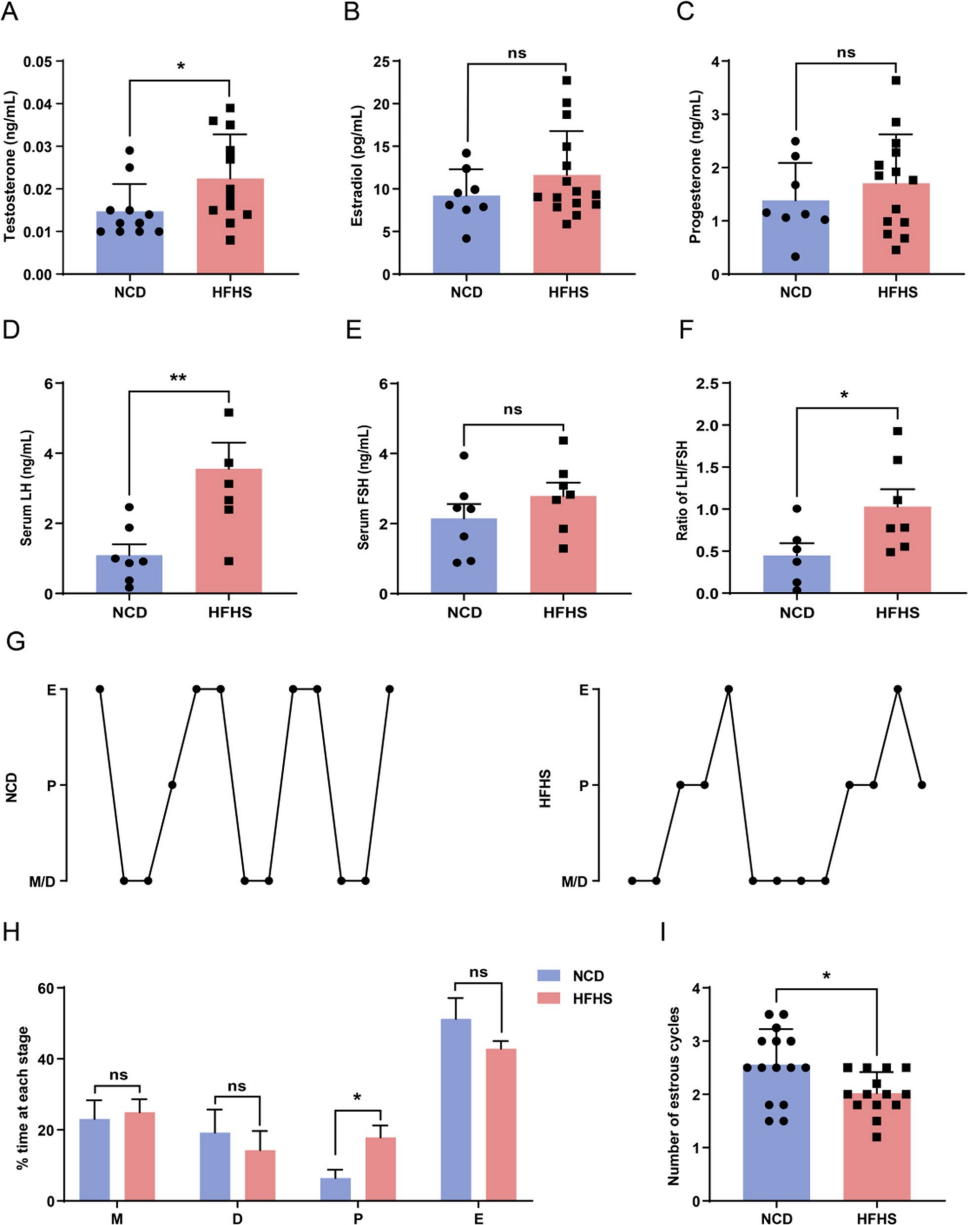
HFHS diet causes hormonal imbalance and irregular estrous cycles in mice. **A** Measurement of serum testosterone (T) levels in serum of control and HFHS mice (control, n = 11; HFHS, n = 12). **B** Measurement of estradiol in serum of control and HFHS mice (control, n = 8; HFHS, n = 15). **C** Measurement of progesterone in serum of control and HFHS mice (control, n = 8; HFHS, n = 15). **D** Serum luteinizing hormone (LH) levels of control and HFHS mice (control, n = 7; HFHS, n = 6). **E** Serum follicle stimulating hormone (FSH) levels of control and HFHS mice (n = 7). **F** Ratio of LH/FSH of control and HFHS mice (control, n = 6; HFHS, n = 7). **G** Representative estrous cycles of control and HFHS mice. Vaginal cytology was assessed for 13 days. M, metestrus; D, diestrus; P, proestrus; E, estrus. **H** Quantitative analysis of estrous cycles in control and HFHS mice (control, n = 6; HFHS, n = 7). **I** The number of total intact cycles during the estrous assessment period (control, n = 15; HFHS, n = 14). Data are presented as mean ± SEM, ns = not statistically significant, *P < 0.05, **P < 0.01

Sexual hormone synthesis and secretion are fundamental functions of the ovary. To determine the impacts of HFHS diet on ovarian function, the serum levels of sexual hormones were measured. The results showed that serum testosterone (T) levels of HFHS mice were significantly elevated than those of control mice (Fig. 3A). Although not statistically significant, levels of estradiol and progesterone were also elevated (Fig. 3B, C). The serum levels of luteinizing hormone (LH) notably increased in HFHS mice, exhibiting a 3.25-fold elevation compared to NCD mice (Fig. 3D), while the follicle stimulating hormone (FSH) levels did not exhibit a significant difference between the two groups (Fig. 3E). Besides, the LH/FSH ratio was significantly higher in the HFHS group compared to the control group, with a fold change of 2.30 (Fig. 3F). It is worth noting that this sexual hormone profile of HFHS mice is similar to the sexual hormones changes observed in PCOS patients, characterized by abnormally elevated T and LH/FSH levels [21].

Further assessment of estrous cycles revealed irregularity in the HFHS mice (Fig. 3G), which was characterized by an increased proestrus ratio, a reduced estrous ratio (Fig. 3H), and a diminished count of total intact estrous cycles (Fig. 3I). The above results confirm the detrimental effects of HFHS diet on female ovarian endocrine function.

### HFHS diet impairs folliculogenesis in female mice

**Fig. 4:**
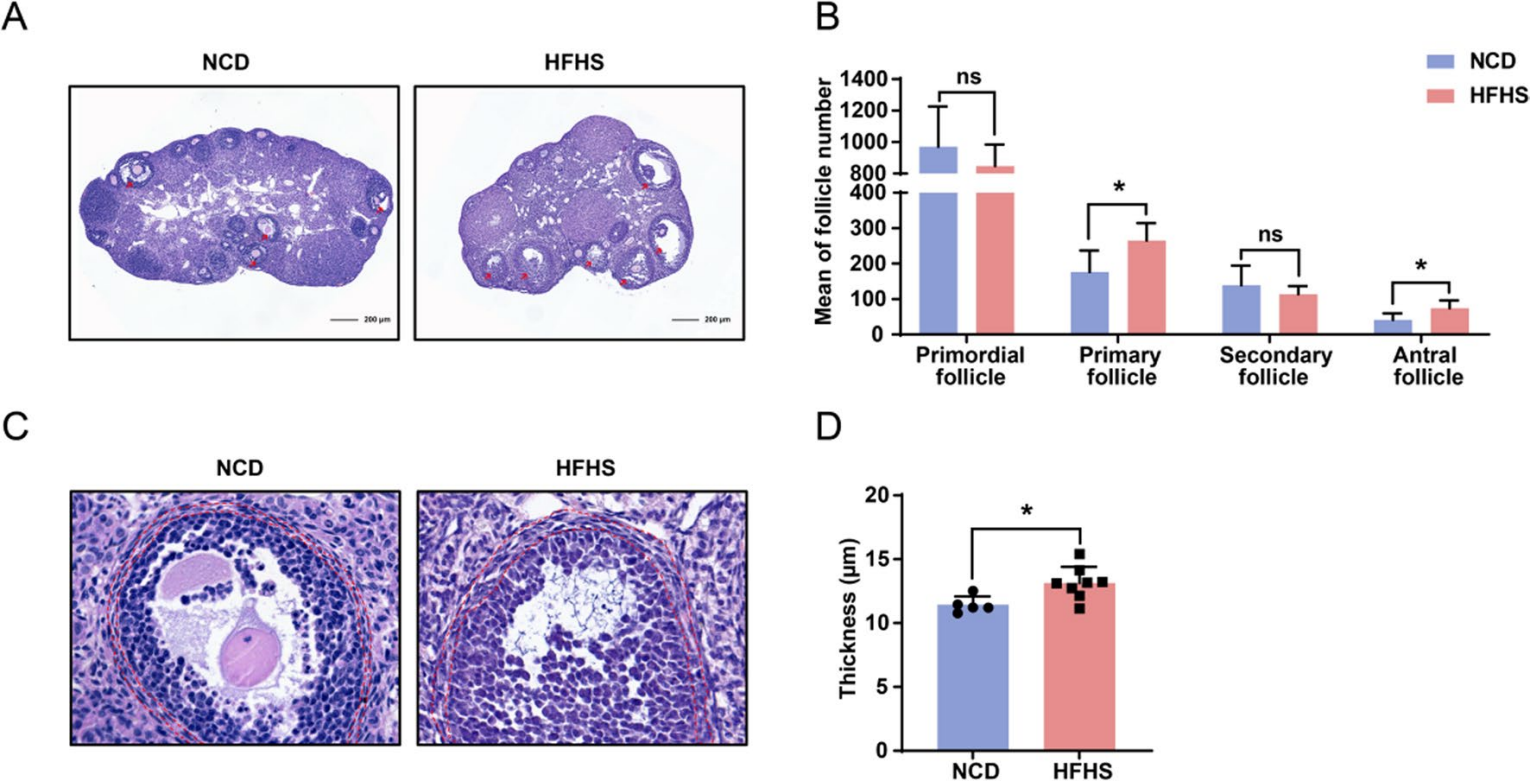
HFHS diet impairs folliculogenesis in female mice. **A** Ovarian morphology of control and HFHS mice. The red arrows indicate antral follicle. **B** Follicle counts of ovaries from control and HFHS mice (control, n = 5; HFHS, n = 8). **C** Representative theca cell layers of antral follicles in control and HFHS mice. **D** Quantitative analysis of thickness of theca cell layers in control and HFHS mice (control, n = 5; HFHS, n = 8). Data are presented as mean ± SEM, ns = not statistically significant, *P < 0.05

To disclose potential morphological changes in the ovaries of HFHS mice, we performed H&E staining with ovarian sections. Despite no obvious differences in ovarian size (Fig. 4A), we noted a pronounced increase in the number of antral follicles in ovaries of HFHS mice. To comprehensively analyze the follicular development, we performed follicle counting using sequential ovarian sections. As shown in Fig. 4B, the number of antral follicles was significantly increased in HFHS mice compared to control mice. Moreover, we observed a significant increase in the number of primary follicles (Fig. 4B).

The ovarian theca cell is the primary cell type responsible for androgen production in females [22]. To investigate the potential involvement of theca cells in the elevated testosterone levels of HFHS mice, we measured the thickness of the ovarian theca cell layers in antral follicles (Fig. 4C). We found that the thickness of the theca cell layer was significantly increased in HFHS mice compared to control mice (Fig. 4D), suggesting hyperplasia of the theca cells in HFHS mice. These results indicate that the folliculogenesis was impaired by HFHS diet in mice, to some extent mirroring the ovarian changes seen in patients with PCOS [23].

### HFHS diet disturbs the ovarian metabolism of female mice revealed by metabolomics

To explore the influence of HFHS diet on ovarian metabolism, a precise metabolomic approach was employed to examine the ovarian metabolic profiles. This approach incorporates a comprehensive set of isotope-labeled standards covering major metabolic pathways for the quantification of polar metabolites, thus greatly improving the quantitative accuracy and precision of metabolite characterization [18]. Then a total of 252 metabolites were detected in the ovaries, classified into 46 classes, including amino acids, glycerophosphocholines, acyl carnitines, and others. Among all the detected metabolites, the most predominant category is amino acids, with 78 metabolites representing 31% of the total metabolites (Additional file 1: Fig. S2A). This metabolomic overview suggests that amino acids may play critical roles in ovarian metabolism.

**Fig. 5:**
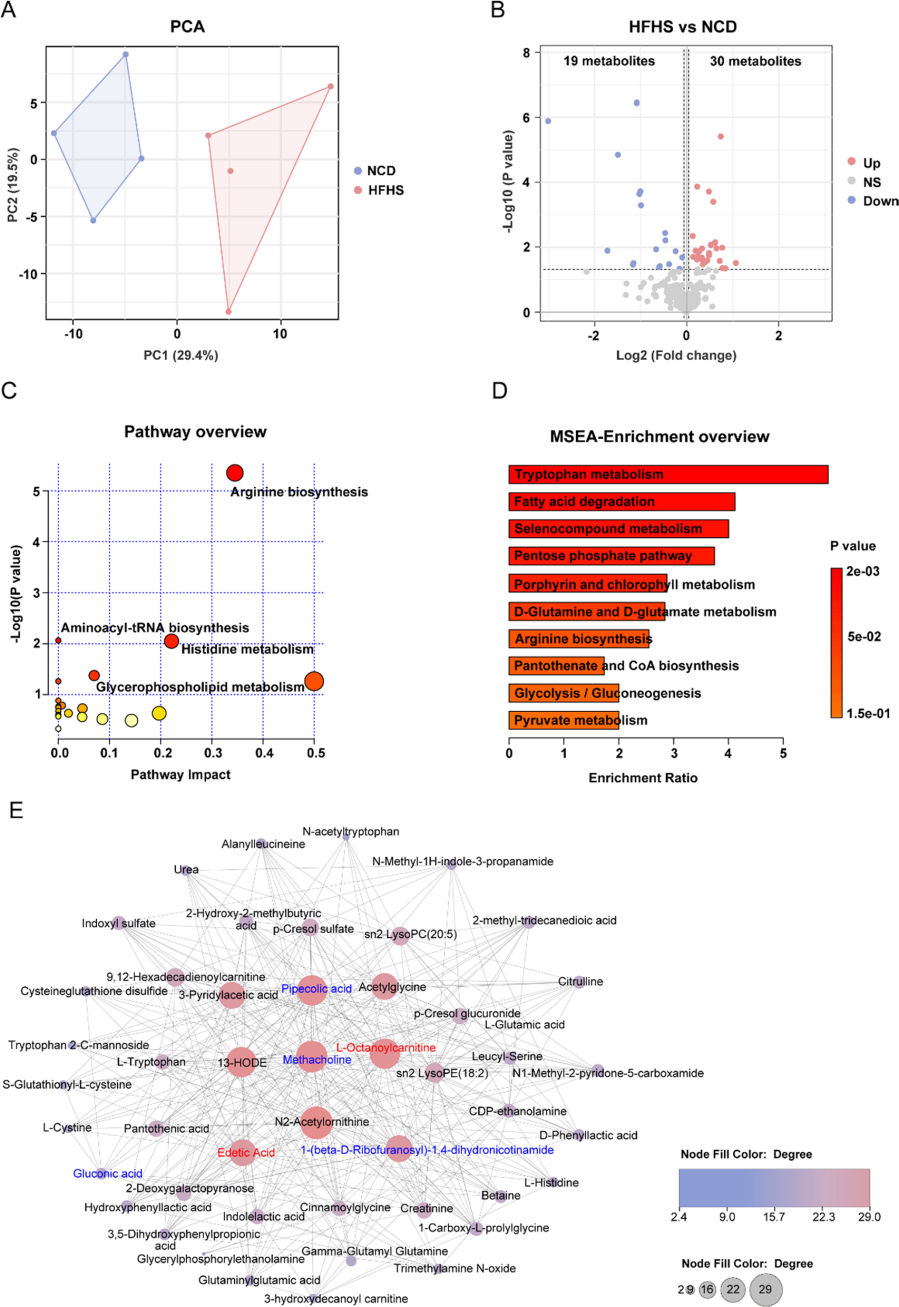
HFHS diet disturbs the ovarian metabolism of female mice revealed by metabolomics. **A** Principal components analysis (PCA) of metabolomics from control and HFHS mice ovaries (n = 4 for each group). **B** Volcano plot of differential metabolites identified in ovary metabolomics. Up-regulated metabolites are shown in red and down-regulated metabolites are shown in blue. **C** Pathway analysis overview of control and HFHS metabolomics. **D** Bar charts of metabolite set enrichment analysis of HFHS group compared to controls. **E** Displaying the differential metabolite network using debiased sparse partial correlation. The bubble size and color represents the significant magnitude of target metabolites enrichment

Principal component analysis (PCA) of the metabolomic data showed a clear distinction between the two groups in PC1, which accounts for 29.4% of the metabolomic variation (Fig. 5A). Further analysis through orthogonal partial least squares discrimination analysis (OPLS-DA) revealed the metabolic deviations between HFHS mice and the control group (Additional file 1: Fig. S2B). Examination of the S-plot identified several phenotypic variables that were highly discriminative for HFHS mice, among which L-lactic acid, oxidized glutathione, and taurocholic acid, which were particularly significant (Additional file 1: Fig. S2C). These findings indicate that substantial metabolic perturbations were instigated in the ovaries of female HFHS mice.

The analysis of differential metabolites identified 49 distinct metabolites in the ovaries of HFHS mice, of which 30 were up-regulated and 19 were down-regulated (Fig. 5B; Additional file 1: Table S1). Pathway enrichment and topology analysis of the 49 differential metabolites revealed several differential metabolic pathways between the HFHS and control ovaries, including arginine biosynthesis, histidine metabolism, aminoacyl-tRNA biosynthesis, and glycerophospholipid metabolism (Fig. 5C). Further metabolite set enrichment analysis revealed significantly enriched metabolic pathways in tryptophan metabolism, fatty acid degradation, selenocompound metabolism, and pentose phosphate pathway (Fig. 5D). Through debiased sparse partial correlation network analysis, we identified several key differential metabolites, including pipecolic acid, methacholine, 1-(beta-d-ribofuranosyl)-1,4-dihydronicotinamide (enriched in arginine biosynthesis), l-octanoylcarnitine, and edetic acid (enriched in glycerophospholipid metabolism and lipid metabolism, Fig. 5E). Taken together, these results suggest that HFHS diet instigates disturbed amino acid and glucolipid metabolism in the ovaries of female mice.

### Transcriptomic analysis reveals HFHS diet-induced dysregulations in ovarian steroid hormone synthesis and glucolipid metabolism

**Fig. 6:**
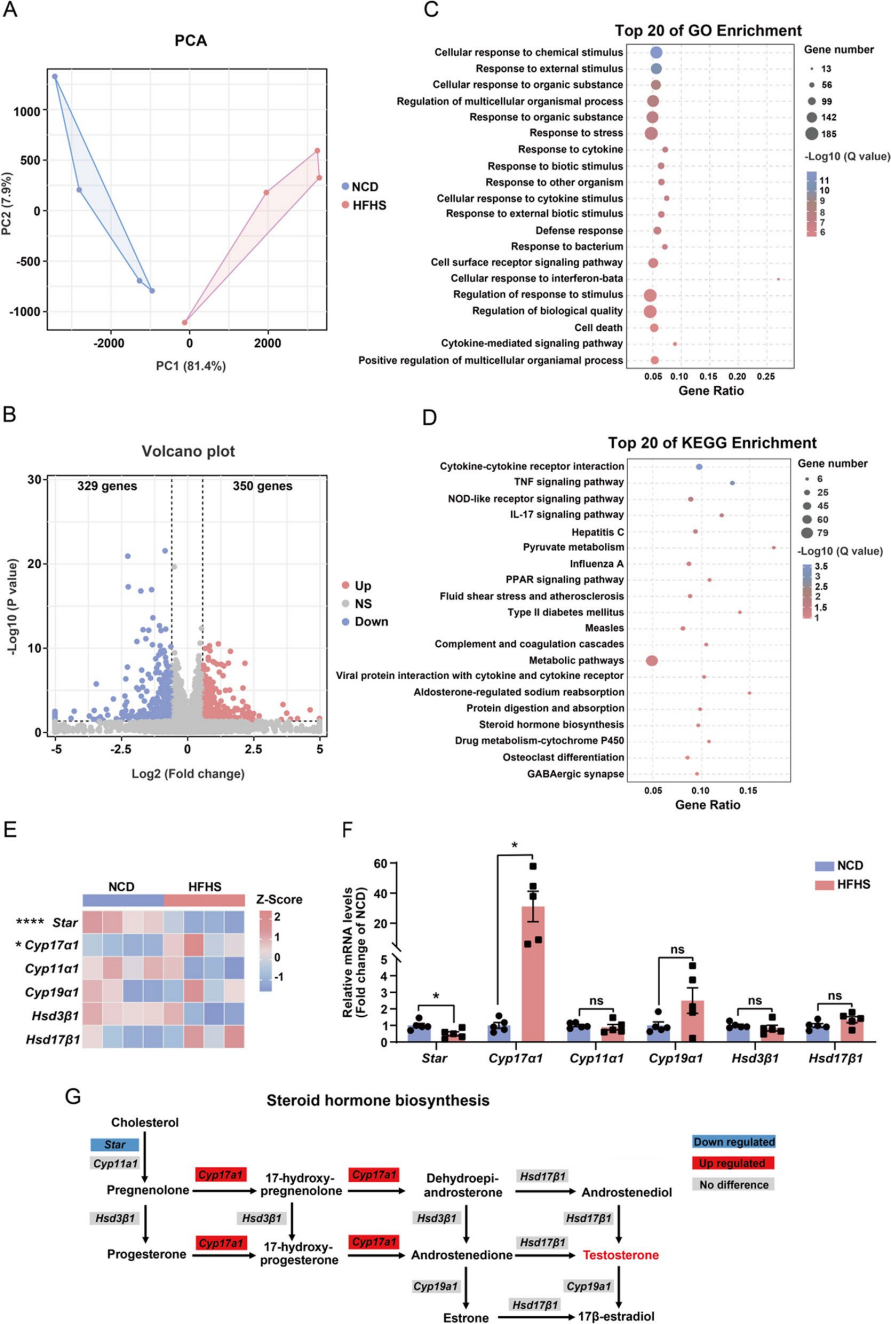
Transcriptomic analysis reveals HFHS diet-induced dysregulations in ovarian steroid hormone synthesis and glucolipid metabolism. **A** PCA of RNA-seq from control and HFHS mice ovaries (n = 4 for each group). **B** Volcano plot of differentially expressed genes (DEGs) identified in ovary RNA-seq. Up-regulated genes are shown in red and down-regulated genes are shown in blue. **C**, **D** Bubble diagrams for the top 20 gene ontology (GO; **C**) and KEGG (**D**) pathway enrichments of the HFHS transcriptomes. The bubble size represents the number of enriched genes, and the bubble color represents the significant magnitude of target gene enrichment. **E** Heatmap of genes involved in ovarian steroid hormone biosynthesis in the RNA-seq data (n = 4). **F** Relative mRNA expression levels of *Star, Cyp17a1, Cyp11a1, Cyp19a1, Hsd3b1, and HSd17b1* in ovaries of control and HFHS mice (n = 5). **G** Schematic diagram illustrating the changes in steroid hormone biosynthesis pathway in HFHS mice. Genes in red rectangular boxes are significantly up-regulated and those in blue boxes are significantly down-regulated in the HFHS transcriptomes. Data are presented as mean ± SEM, ns = not statistically significant, *P < 0.05, ****P < 0.0001

To gain insights into the molecular mechanisms underlying the HFHS-induced ovarian dysfunction, we profiled the transcriptomes of ovaries from mice fed either an NCD or HFHS diet. PCA of the RNA sequencing (RNA-seq) data showed a clear distinction between the two groups (Fig. 6A). Differential gene expression analysis identified 350 up-regulated genes and 329 down-regulated genes in HFHS group compared with the NCD group (Fig. 6B). By performing gene ontology (GO) enrichment analysis of the differentially expressed genes (DEGs), we found that pathways of immune activation and inflammatory response were significantly enriched (Fig. 6C). KEGG-based pathway analysis revealed a significant enrichment in immunological pathways and metabolic pathways, including pyruvate metabolism, PPAR signaling pathway, and type 2 diabetes mellitus (Fig. 6D). To be noted, the steroid hormone biosynthesis pathway was significantly enriched in the HFHS ovarian transcriptome (Fig. 6D), which could account for the abnormal sexual hormone levels in these mice (Fig. 3A).

To investigate the common trends of changes in the ovarian gene set, we performed gene set enrichment analysis (GSEA) of the ovarian transcriptomes. The results revealed several up-regulated pathways, including steroid receptor-related pathways, PI3K-Akt signaling pathway, estrogen signaling pathway, apoptosis-related pathways, and TNF signaling pathway (Additional file 1: Fig. S3A–F). The down-regulated GSEA pathways included pyruvate and lipid metabolism, circadian rhythm, and oxidative phosphorylation etc. (Additional file 1: Fig. S3G–L).

Based on the observation that steroid hormone synthesis pathway was enriched in the HFHS ovarian transcriptome, we further examined the expression levels of ovarian steroid synthesis-related pivotal genes. The results showed that *Cyp17a1*, the key enzyme responsible for androgen synthesis possessing both 17alpha-hydroxylase and 17,20-lyase activities, was significantly up-regulated in the ovaries of HFHS mice (Fig. 6E, F). Whereas *Star*, the enzyme that is responsible for pregnenolone synthesis, was significantly down-regulated in HFHS ovaries (Fig. 6E, F). The combined expression alterations of these two essential genes involved in steroid hormone synthesis could elucidate the augmented production of testosterone (Fig. 6G). Collectively, the aforementioned data indicate that HFHS diet disrupts the expression of genes implicated in ovarian steroid synthesis and glucolipid metabolism, leading to the development of female reproductive disorders.

### Integrated metabolomic and transcriptomic analyses unveil aberrations in ovarian arginine metabolism induced by HFHS diet

**Fig. 7:**
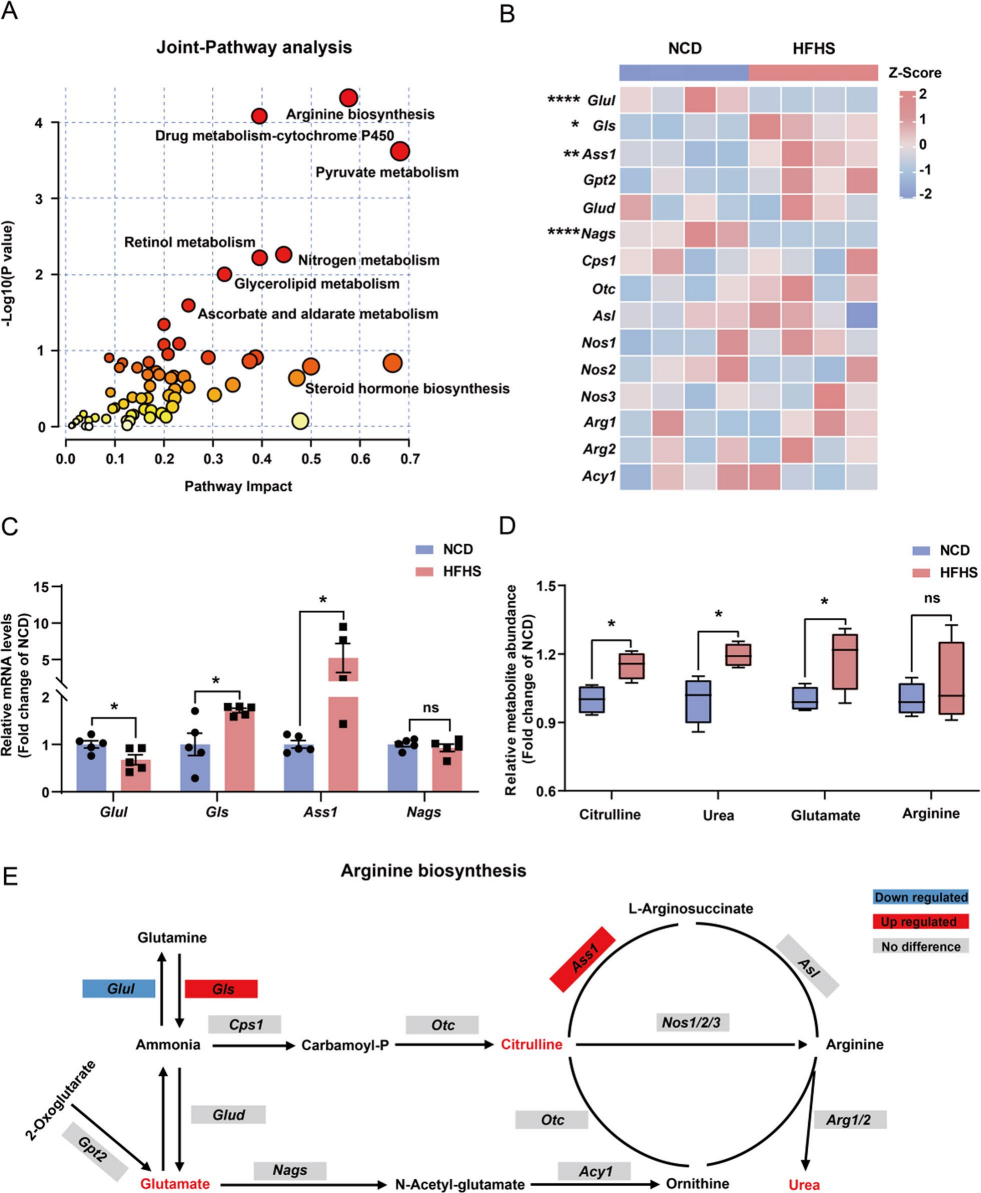
Integrated metabolomic and transcriptomic analyses unveil aberrations in ovarian arginine metabolism induced by HFHS diet. **A** Integrated analyses of differential metabolites and RNA-seq DEGs by joint-pathway analysis. **B** Heatmap of genes involved in arginine biosynthesis in the RNA-seq data (n = 4). **C** Relative mRNA expression levels of *Glul, Gls, Ass1* and *Nags* in ovaries from control and HFHS mice (n = 5). **D** Relative levels of differential metabolites in the arginine biosynthetic pathway (citrulline, urea, glutamate, and arginine, n = 4). **E** Schematic diagram illustrating the alterations in the arginine biosynthesis pathway in HFHS ovaries. Genes enclosed in red rectangular boxes indicate significant up-regulation, while the genes in the blue boxes represent significant down-regulation. Additionally, metabolites shown in red font indicate a significant increase in the HFHS metabolomes. Data are presented as mean ± SEM, ns = not statistically significant, *P < 0.05, **P < 0.01, ****P < 0.0001

To further dissect the molecular and metabolic signatures of ovaries from HFHS mice, we performed combined analyses of the transcriptomic and metabolomic data. The joint-pathway results showed enrichments of pyruvate metabolism, nitrogen metabolism, glycerolipid metabolism, and steroid hormone biosynthesis pathways (Fig. 7A). Notably, arginine biosynthesis was found to be the most significantly enriched pathway (Fig. 7A). We found that the mRNA expression levels of *Gls* (encoding an enzyme that catalyzes the hydrolysis of glutamine to produce ammonia and glutamate)*,* and *Ass1* (encoding a key enzyme in arginine biosynthesis that catalyzes the formation of argininosuccinate from citrulline) were significantly increased in HFHS ovaries (Fig. 7B, C). In contrast, the mRNA expression levels of *Glul*, which catalyzes the synthesis of glutamine from glutamate and ammonia in an ATP-dependent reaction, was down-regulated (Fig. 7B, C). Consistently, the metabolites involved in arginine metabolism, including glutamate, citrulline, and urea, were also markedly up-regulated in the ovaries of HFHS mice (Fig. 7D).

As shown in Fig. 7E, the combined effects of increased *Gls* expression and decreased *Glul* expression in HFHS ovaries could enhance the synthesis of ammonia and glutamate from glutamine. Given the absence of observed ammonia accumulation in the HFHS ovaries (Additional file 1: Table S1), it is plausible that the downstream flux from ammonia to citrulline metabolism is elevated, as evidenced by the markedly elevated level of citrulline (Fig. 7D). Furthermore, the significantly up-regulated *Ass1* could facilitate the synthesis of urea from citrulline (Fig. 7E). Taken together, the above results suggest that HFHS diet alters the expression of genes essential for arginine biosynthesis, culminating in the accumulation of corresponding metabolites, which might contribute to ovarian dysfunction.

### HFHS mice phenocopy the reproductive and metabolic signatures of women with PCOS

**Fig. 8:**
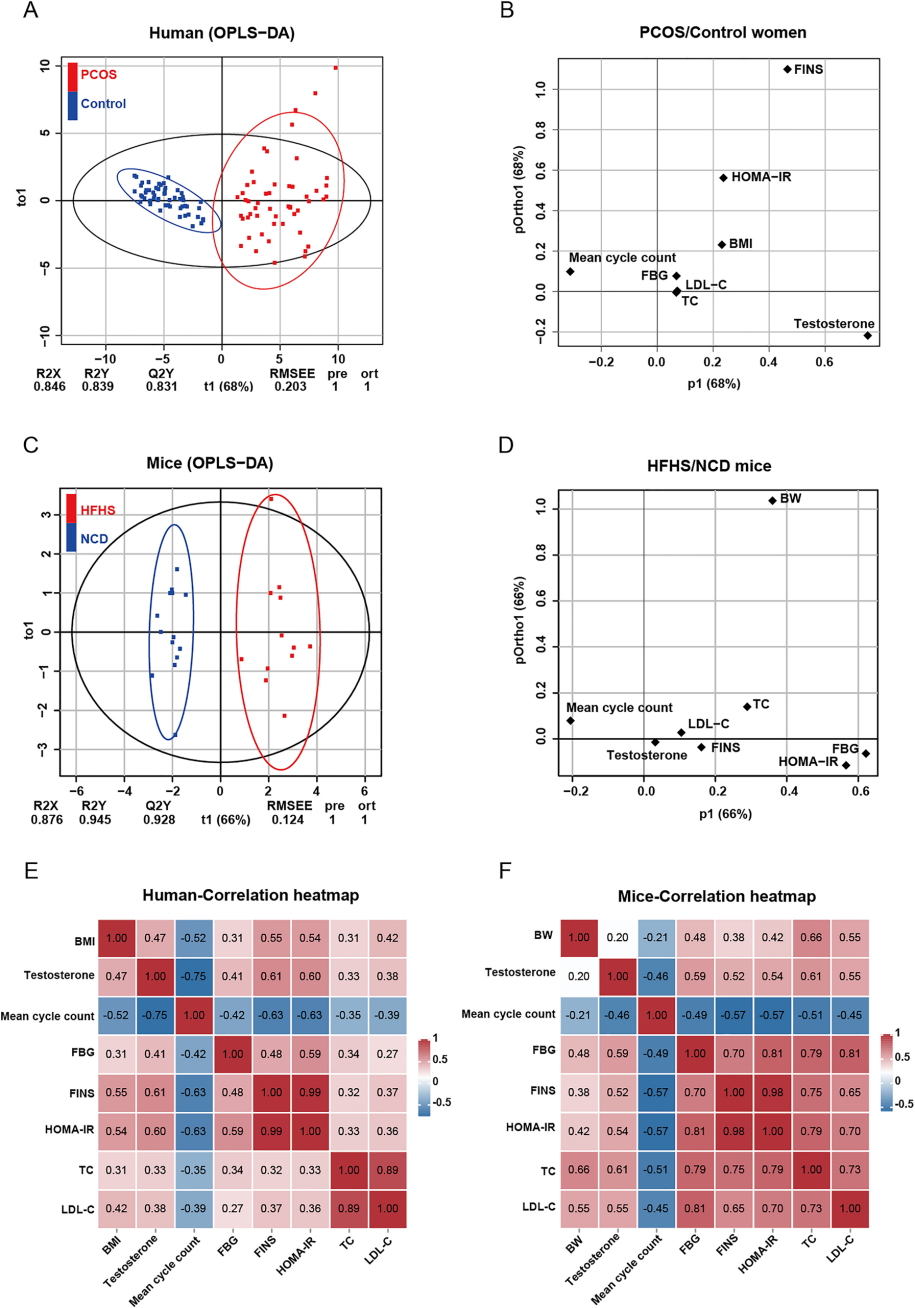
HFHS mice phenocopy the reproductive and metabolic signatures of women with PCOS. **A** The score plot of  OPLS-DA depicts women with PCOS (n = 64, represented by red dots) and control women (n = 68, represented by blue dots). (B) Comparison of OPLS-DA load plots from PCOS and control women based on 8 reproductive or metabolic criteria. **C** OPLS-DA score plots for HFHS (n = 12, red dots) and NCD (n = 14, blue dots) mice. **D** Comparison of OPLS-DA load plots from control and HFHS mice based on 8 reproductive or metabolic criteria. **E**–**F** Pearson’s correlation matrix between the reproductive and metabolic parameters for women subjects (**E**) and mice (**F**). The values plotted in the graph represent the correlating coefficients

Given the signs of hyperandrogenism, increased ovarian follicles, obesity, and insulin resistance in female HFHS mice, which are classic features of women with PCOS [24], we sought to investigate whether this HFHS mouse could simulate obese women with PCOS. For this purpose, a cohort of overweight women diagnosed with PCOS were enrolled according to the Rotterdam criteria. These patients presented typical PCOS phenotypes, including hyperandrogenism, menstrual disturbance, polycystic ovarian morphology, as well as metabolic dysfunctions of insulin resistance and dyslipidemia (Additional file 1: Table S2). We developed a multivariate model using the OPLS-DA method to compare the clinical characteristics of PCOS women with controls. By using this method, a clear distinction between PCOS women and controls were detected (Fig. 8A). The Q2Y and R2Y values of OPLS-DA were 0.831 and 0.839, respectively (Fig. 8A), indicating strong validity and predictive ability to accurately discriminate between PCOS and control groups. The clinical parameters for this model construction included fasting insulin (FINS), HOMA-IR, LDL-C, TC, FBG, body mass index (BMI), testosterone, and mean cycle count (Fig. 8B).

**Fig. 9:**
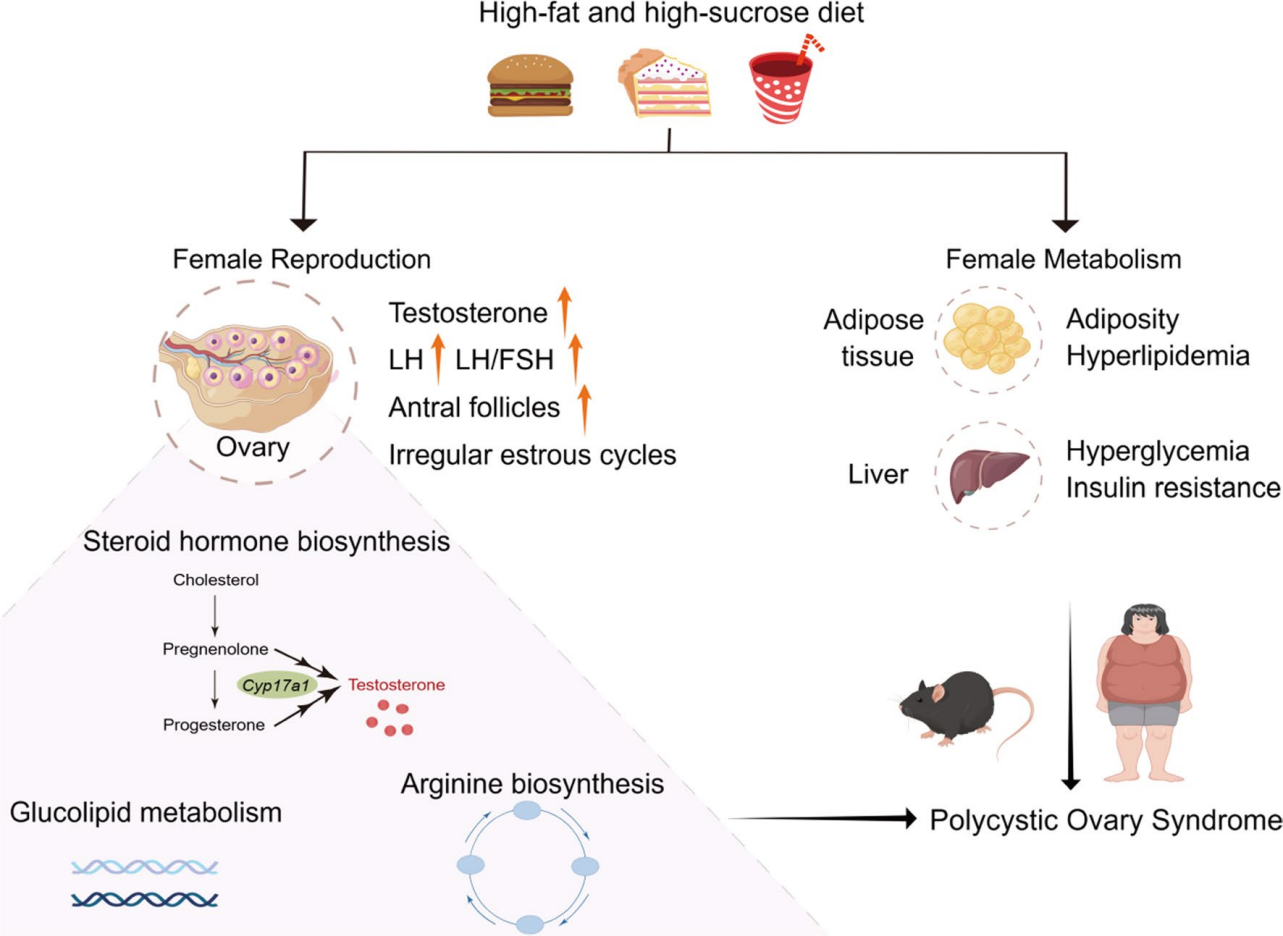
Schematic diagram depicting the role of HFHS diet in female reproductive disorders. Proposed working model of HFHS diet-induced female reproductive and metabolic disorders. The HFHS diet induces heightened levels of serum testosterone, LH, and LH/FSH ratio, as well as an increase in ovarian antral follicles and irregular estrous cycles in female mice. Mechanistically, the HFHS diet disrupts the steroid hormone biosynthesis, glucolipid metabolism, and arginine biosynthesis pathways in the ovary. Furthermore, metabolic dysfunctions, including adiposity, hyperglycemia, and insulin resistance, are observed in HFHS mice. Collectively, these reproductive and metabolic abnormalities contribute to the onset of polycystic ovary syndrome

By utilizing the OPLS-DA method and metrics used in the aforementioned human PCOS cohort data, we established a multivariate model to compare the characteristics of HFHS females with those of the control group. We found that HFHS diet-induced mice were clearly distinguishable from control mice (Fig. 8C, D). This suggests that the HFHS mouse model closely mirrors human PCOS in terms of the above clinical parameters. Using Pearson’s correlation test, a significantly negative correlation was observed between the mean cycle count and serum testosterone levels in women subjects, as was also the case for the correlation between mean cycle count and metabolic indices such as FBG, FINS, etc. (Fig. 8E). Furthermore, significantly positive correlations were observed between serum testosterone levels and FBG, FINS, HOMA-IR, or TC in HFHS mice (Fig. 8F), paralleling the observations in women with PCOS (Fig. 8E), suggesting the HFHS mice and women with PCOS share similar reproductive and metabolic signatures. Taken together, the HFHS diet, reflective of overnutrition and recognized to induce multiple metabolic disorders, can also provoke female reproductive dysfunction and the onset of PCOS (Fig. 9).

## Discussion

In the present study, we found that female mice subjected to a high-fat and high-sucrose (HFHS) diet exhibited both reproductive and metabolic disturbances. Specifically, the HFHS mice displayed phenotypes of obesity, dyslipidemia, hyperglycemia, and insulin resistance. Moreover, these HFHS mice showed significantly elevated serum testosterone and LH levels, irregular estrous cycles, as well as increased numbers of primary and antral follicles, mimicking the clinical manifestations of women with polycystic ovarian syndrome (PCOS). Further integrated analyses of ovarian transcriptome and metabolome revealed profound aberrations in steroid hormone synthesis and amino acid metabolism, especially the arginine biosynthesis pathway. Notably, we identified analogous reproductive and metabolic signatures shared between HFHS mice and PCOS women (Fig. 9). These findings not only provide compelling evidence that the HFHS diet can induce female reproductive disorders but also present a rodent model of PCOS that fully captures the reproductive and metabolic hallmarks of PCOS for pathogenesis investigation.

In recent years, several findings have highlighted the metabolic dysfunction as an underlying mechanism in the pathogenesis of PCOS [25]. Although hyperandrogenism has been considered the main cause of PCOS, animal models constructed by androgen administration have failed to encompass the full spectrum of PCOS clinical features, including reproductive, endocrine, and metabolic abnormalities [26]. For instance, prenatal dihydrotestosterone exposure could effectively emulate the PCOS characteristics such as hyperandrogenemia, disrupted menstrual cycles, and aberrant follicular development [27]. However, the metabolic phenotypes of these mice are mild, without any discernible alterations in body weight and adiposity [28]. This prenatal androgen model mimics a lean phenotype of PCOS, whereas clinically PCOS women often manifest obesity and insulin resistance. In addition, DHEA treatment alone induced unstable phenotypes in mice, with both obvious and negligible metabolic derangements being reported [28, 29]. By contrast, the HFHS mouse model not only displays PCOS-like reproductive abnormalities such as hyperandrogenemia, increased LH levels, irregular estrous cycles, and impaired folliculogenesis but also exhibits strong metabolic disorders, including glucose intolerance, insulin resistance, hyperlipidemia, and hepatic steatosis. During the development of PCOS, metabolic and reproductive abnormalities are intricately interacted [30]. For example, the excess of glucose and lipids can not only lead to insulin resistance and type 2 diabetes [31, 32] but also induce ovarian inflammation in PCOS women [33]. In addition, insulin resistance and compensatory hyperinsulinemia in obese women can further exacerbate hyperandrogenism [34, 35]. Therefore, the HFHS diet-induced hyperglycemia, dyslipidemia, and insulin resistance could all serve as driving factors for the onset of PCOS.

Since PCOS is a complex reproductive metabolic syndrome influenced by both genetic and environmental factors [21], interventions that focus solely on single symptom of PCOS have limited efficacy in the management of this disease [36, 37]. A comprehensive understanding of the altered metabolic milieu of PCOS women is essential to improve its prevention and management [38]. To investigate the molecular mechanism underlying HFHS-induced reproductive dysfunction, we performed metabolomic and transcriptomic analyses on ovaries from the HFHS and control mice, revealing remarkable disturbances in amino acid metabolic pathways. Interestingly, the correlations between PCOS and amino acid metabolism have been reported in several clinical investigations. Perturbations in the tryptophan metabolism pathway, enriched in ovarian metabolomes of HFHS mice, have also been identified in metabolomics analyses of follicular fluid [39], urine [40], and plasma [41, 42] of PCOS patients. In addition, the differential metabolites such as citrulline and cystine identified in HFHS ovaries were also enriched in the plasma metabolomics of PCOS patients [43, 44]. The above clinical evidence, combined with our observations in HFHS rodents, underscores the disrupted amino acid metabolism as a crucial metabolic signature in PCOS.

Utilizing integrated transcriptomic and metabolomic analyses, we further identified arginine biosynthesis pathway as the most prominent alteration in the ovaries of HFHS mice. The levels of citrulline, glutamate, and urea, which are involved in this pathway, were found to be markedly increased in the ovaries of the HFHS mice. However, the arginine abundance showed no significant change. Since both citrulline and urea, as the source and downstream metabolites of arginine, are increased in the *Ass1*-driven urea cycle, this increased flux of urea cycle may account for the insignificant change in the abundance of arginine itself. Therefore, measurement of the urea cycle flux and identifying its impact on female reproductive disorders such as PCOS deserve future investigation. Besides, whether arginine is metabolized by other pathways is unknown. Intriguingly, arginine biosynthesis pathway has been found to be significantly enriched in various metabolomics studies conducted in plasma [43], fecal [45], and follicular fluid [39] from PCOS patients. The close associations between arginine pathway and PCOS have been recognized, revealing altered plasma levels of nitric oxide and arginine-related transcripts in PCOS women [46]. A clinical study also investigated the potential use of arginine as a supplementary medication for PCOS treatment [47]. However, the exact contribution of this pathway to PCOS etiology remains to be elucidated. Future functional studies are needed to identify the causal effects of arginine biosynthesis pathway in the development of PCOS. Therefore, these ovarian multi-omics data from the HFHS mice could serve as unique resources and original tools to dissect the molecular mechanisms in the pathogenesis of PCOS and female infertility.

Taken together, the present study provides compelling in vivo evidence that an HFHS diet could trigger female reproductive dysfunction and the onset of PCOS. These insights bolster the notion of a metabolic origin for PCOS, highlighting the critical role of high fat combined with high sucrose intake in the development of this disease. As such, lifestyle interventions, including low-carbohydrate or calorie-restricted diets, could be promising approaches for effectively managing PCOS or other female reproductive diseases associated with metabolic disorders. Furthermore, our study provides a detailed molecular and metabolic landscape of ovaries impacted by an HFHS diet, paving the way for future mechanistic investigations and the development of innovative therapeutic strategies for female reproductive diseases.

## Conclusions

Our study establishes that an HFHS diet of overnutrition could cause detrimental effects on female reproduction and metabolism, leading to the development of PCOS. These results highlight the need for dietary modifications in safeguarding women’s reproductive health as well as effectively managing PCOS.

### Supplementary Information

**Additional file 1: Figure S1. **Quantification of adipocytes size. **Figure S2.** Overview of ovarian metabolomic in HFHS-treated mice compared to controls.** Figure S3.** Gene set enrichment analysis of ovarian transcriptomic in HFHS-treated mice compared to controls. **Table S1.** List of differential metabolites in ovaries of HFHS-treated mice compared to controls. **Table S2.** Clinical characteristics of control and PCOS subjects. **Table S3. **Sequences of primers.

## Data Availability

All data generated for this study can be accessed from the corresponding author upon reasonable request. The RNA sequencing data files have been deposited in the Gene Expression Omnibus (GEO) database under the accession code GSE240547.

## Abbreviations

HFHS: High-fat and high-sucrose
PCOS: Polycystic ovary syndrome
T2D: Type 2 diabetes
NCD: Normal chow diet
TC: Total cholesterol
LDL-C: Low-density lipoprotein cholesterol
HDL-C: High-density lipoprotein cholesterol
TG: Triglyceride
H&E: Hematoxylin and eosin
SAT: Subcutaneous white adipose tissue
VAT: Visceral white adipose tissue
ORO: Oil Red O
FBG: Fasting blood glucose
GTT: Glucose tolerance test
AUC: Area under the curve
ITT: Insulin tolerance test
T: Testosterone
E_2_: Estradiol
LH: Luteinizing hormone
FSH: Follicle stimulating hormone
PCA: Principal component analysis
OPLS-DA: Orthogonal partial least squares discrimination analysis
RNA-seq: RNA sequencing
GO: Gene ontology
DEGs: Differentially expressed genes
GSEA: Gene set enrichment analysis
FINS: Fasting insulin
BMI: Body mass index
