# Supplementary material for: High-fat and high-sucrose diet impairs female reproduction by altering ovarian transcriptomic and metabolic signatures
Source: J Transl Med. 2024 Feb 12;22:145. doi: 10.1186/s12967-024-04952-y (PMC10860219; doi:10.1186/s12967-024-04952-y)
Supplement: Supplementary file 1 — Additional file 1: Figure S1. Quantification of adipocytes size. Figure S2. Overview of ovarian metabolomic in HFHS-treated mice compared to controls. Figure S3. Gene set enrichment analysis of ovarian transcriptomic in HFHS-treated mice compared to controls. Table S1. List of differential metabolites in ovaries of HFHS-treated mice compared to controls. Table S2. Clinical characteristics of control and PCOS subjects. Table S3. Sequences of primers. [file 12967_2024_4952_MOESM1_ESM.docx]

**Additional file 1**

**High-fat and high-sucrose diet impairs female reproduction by altering ovarian transcriptomic and metabolic signatures**

**
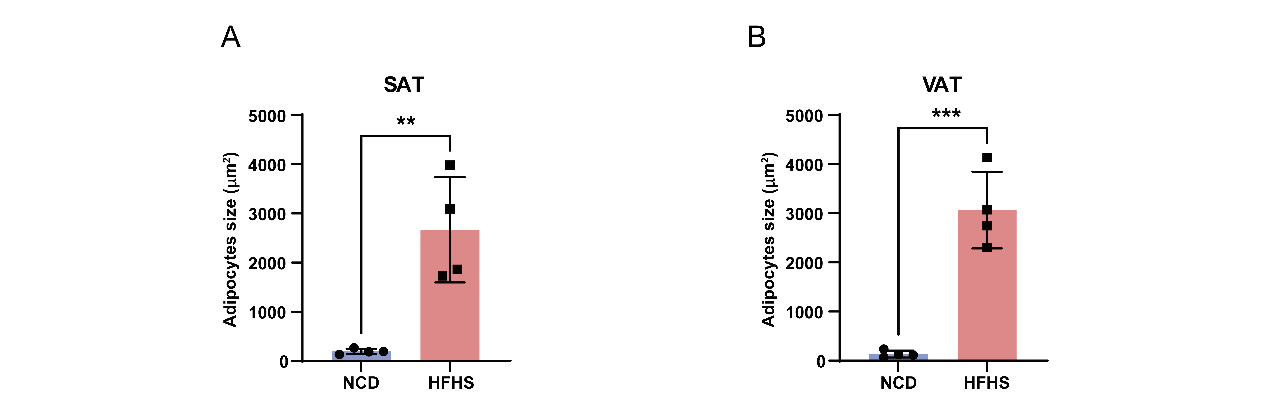
**

**Figure S1: Quantification of adipocytes size.**

1. Quantification of adipocytes size in subcutaneous white adipose tissue (SAT) of control and HFHS mice (n = 4). (B) Quantification of adipocytes size in visceral white adipose tissue (VAT) of control and HFHS mice (n = 4). Data are presented as mean ± SEM, ***P* < 0.01, ****P* < 0.001.


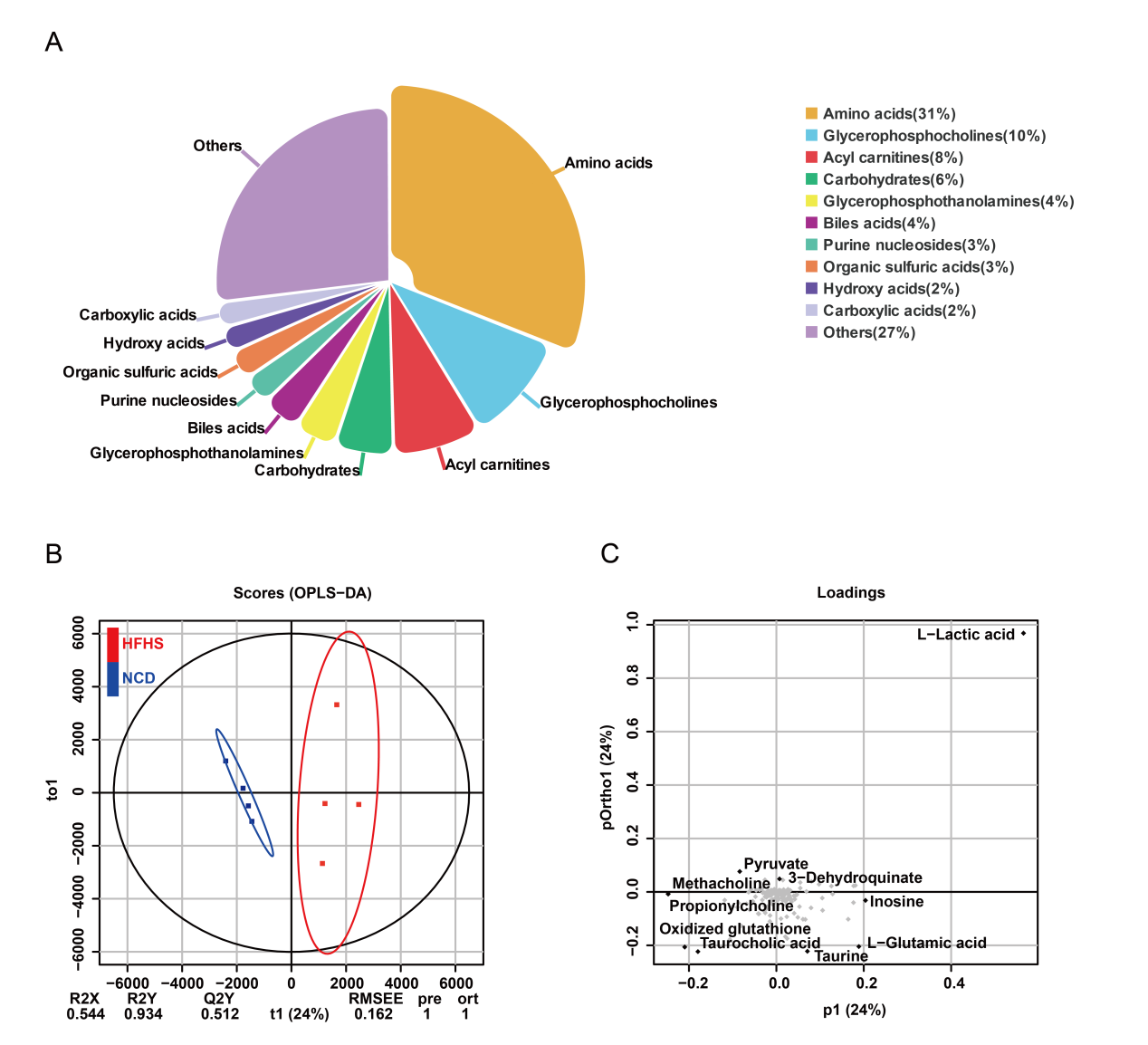


**Figure S2: Overview of ovarian metabolomic in HFHS-treated mice compared to controls.**

1. The pie chart shows a detailed breakdown of the average percentage distribution of ovarian metabolites, “others” represents the percentage of the remaining 36 metabolite classes. (B) The score plot of orthogonal partial least squares discriminant analysis (OPLS-DA) depicts control (n = 4, represented by blue dots) and HFHS mice (n = 4, represented by red dots). (C) Comparison of OPLS-DA load plots from control and HFHS mice (n = 4).


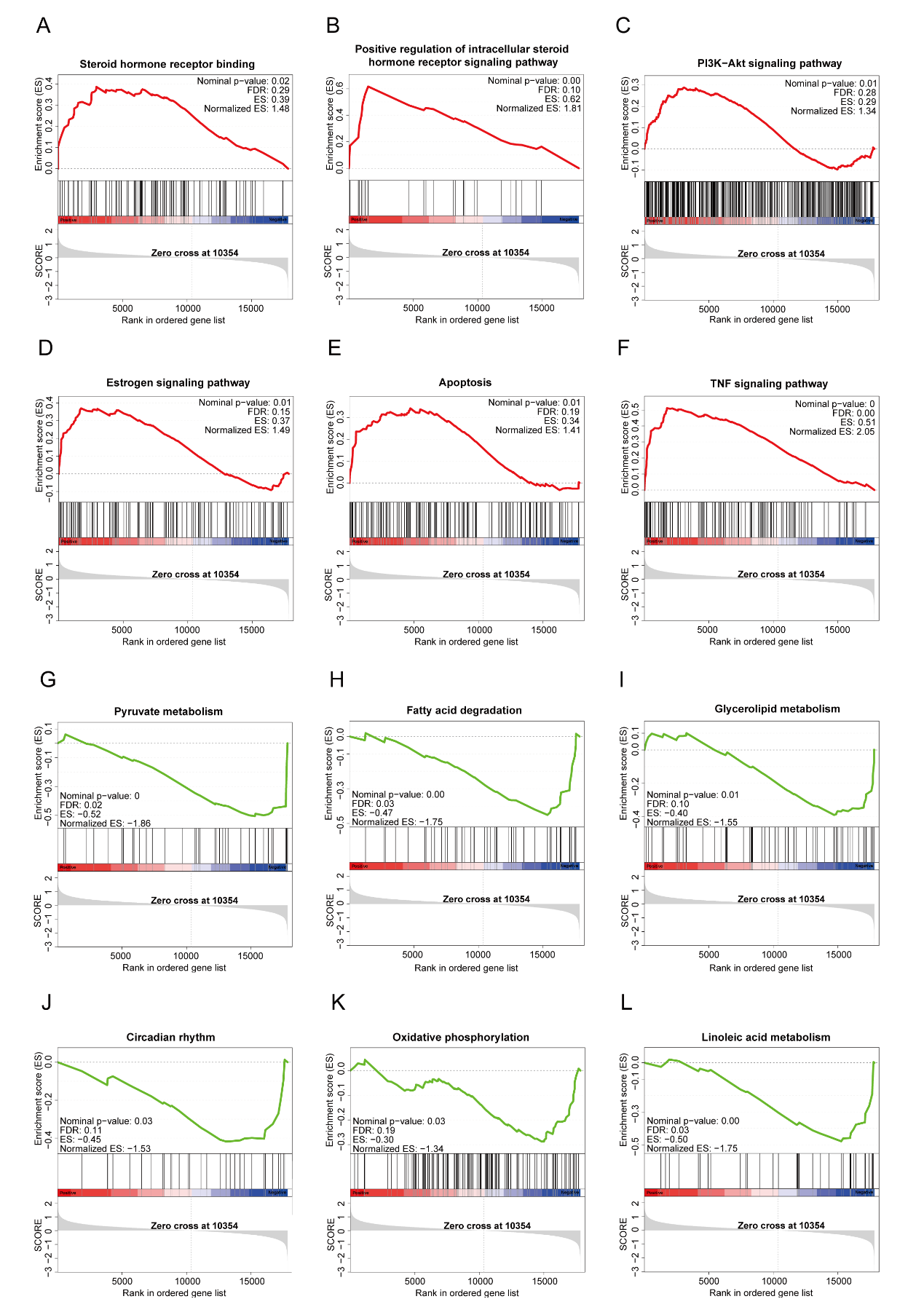


**Figure S3: Gene set enrichment analysis of ovarian transcriptomic in HFHS-treated mice compared to controls.**

1. GSEA analysis of steroid hormone receptor binding. (B) GSEA analysis of positive regulation of intracellular steroid hormone receptor signaling pathway. (C) GSEA analysis of PI3K-Akt signaling pathway. (D) GSEA analysis of estrogen signaling pathway. (E) GSEA analysis of apoptosis. (F) GSEA analysis of TNF signaling pathway. (G) GSEA analysis of pyruvate metabolism. (H) GSEA analysis of fatty acid degradation. (I) GSEA analysis of glycerolipid metabolism. (J) GSEA analysis of circadian rhythm. (K) GSEA analysis of oxidative phosphorylation. (L) GSEA analysis of linoleic acid metabolism.

**Table S1: List of differential metabolites in ovaries of HFHS-treated mice compared to controls.**

|  | Metabolite Name | Fold change | *P* value | VIP |
| --- | --- | --- | --- | --- |
| 1 | Propionylcholine | 0.47 | <0.001 | 1.97 |
| 2 | Methacholine | 0.47 | <0.001 | 1.97 |
| 3 | N2-Acetylornithine | 0.12 | <0.001 | 1.97 |
| 4 | Pantothenic acid | 1.68 | <0.001 | 1.95 |
| 5 | 13-HODE | 0.36 | <0.001 | 1.95 |
| 6 | L-Tryptophan | 1.18 | <0.001 | 1.87 |
| 7 | 3-Pyridylacetic acid | 0.50 | <0.001 | 1.91 |
| 8 | 2-Hydroxy-2-methylbutyric acid | 1.41 | <0.001 | 1.87 |
| 9 | Pipecolic acid | 0.49 | <0.001 | 1.90 |
| 10 | 2-Deoxygalactopyranose | 1.50 | <0.001 | 1.83 |
| 11 | Edetic Acid | 0.51 | <0.001 | 1.85 |
| 12 | 1-(beta-D-Ribofuranosyl)-1,4-dihydronicotinamide | 0.73 | <0.001 | 1.74 |
| 13 | Creatinine | 1.10 | <0.001 | 1.70 |
| 14 | Acetylglycine | 0.73 | 0.01 | 1.70 |
| 15 | Indolelactic acid | 1.55 | 0.01 | 1.74 |
| 16 | Cinnamoylglycine | 1.54 | 0.01 | 1.73 |
| 17 | Hydroxyphenyllactic acid | 1.45 | 0.01 | 1.72 |
| 18 | 3,5-Dihydroxyphenylpropionic acid | 1.45 | 0.01 | 1.72 |
| 19 | p-Cresol glucuronide | 1.72 | 0.01 | 1.63 |
| 20 | Indoxyl sulfate | 1.58 | 0.01 | 1.63 |
| 21 | 2-methyl-tridecanedioic acid | 1.27 | 0.01 | 1.60 |
| 22 | L-Octanoylcarnitine | 0.63 | 0.01 | 1.62 |
| 23 | Trimethylamine N-oxide | 0.31 | 0.01 | 1.66 |
| 24 | Citrulline | 1.15 | 0.01 | 1.60 |
| 25 | L-Histidine | 1.22 | 0.01 | 1.56 |
| 26 | Gluconic acid | 0.85 | 0.01 | 1.64 |
| 27 | Urea | 1.19 | 0.01 | 1.59 |
| 28 | S-Glutathionyl-L-cysteine | 1.41 | 0.02 | 1.63 |
| 29 | Cysteineglutathione disulfide | 1.41 | 0.02 | 1.60 |
| 30 | Betaine | 1.19 | 0.02 | 1.58 |
| 31 | Tryptophan 2-C-mannoside | 1.11 | 0.02 | 1.60 |
| 32 | CDP-ethanolamine | 0.94 | 0.02 | 1.50 |
| 33 | Glutaminylglutamic acid | 1.27 | 0.02 | 1.54 |
| 34 | Gamma-Glutamyl Glutamine | 1.26 | 0.02 | 1.52 |
| 35 | N1-Methyl-2-pyridone-5-carboxamide | 1.17 | 0.02 | 1.53 |
| 36 | 1-Carboxy-L-prolylglycine | 1.37 | 0.03 | 1.52 |
| 37 | Alanylleucineine | 1.66 | 0.03 | 1.52 |
| 38 | N-acetyltryptophan | 1.32 | 0.03 | 1.47 |
| 39 | sn2 LysoPC(20:5) | 0.45 | 0.03 | 1.43 |
| 40 | p-Cresol sulfate | 2.11 | 0.03 | 1.54 |
| 41 | N-Methyl-1H-indole-3-propanamide | 0.77 | 0.03 | 1.43 |
| 42 | D-Phenyllactic acid | 1.28 | 0.03 | 1.49 |
| 43 | 9,12-Hexadecadienoylcarnitine | 0.45 | 0.04 | 1.47 |
| 44 | 3-hydroxydecanoyl carnitine | 0.67 | 0.04 | 1.47 |
| 45 | sn2 LysoPE(18:2) | 0.66 | 0.04 | 1.38 |
| 46 | Leucyl-Serine | 1.72 | 0.05 | 1.43 |
| 47 | L-Cystine | 1.80 | 0.05 | 1.46 |
| 48 | Glycerylphosphorylethanolamine | 0.90 | 0.05 | 1.45 |
| 49 | L-Glutamic acid | 1.18 | 0.05 | 1.32 |

**Table S2: Clinical characteristics of control and PCOS subjects.**

|  | Control women  (n = 68) | PCOS women  (n = 64) | *P* value |
| --- | --- | --- | --- |
| Age (years) | 29.16 ± 3.59 | 28.50 ± 3.23 | 0.27 |
| BMI (kg/m2) | 22.34 ± 2.67 | 26.76 ± 3.97 | <0.001 |
| Testosterone (ng/dL) | 25.83 ± 8.45 | 52.65 ± 8.70 | <0.001 |
| Estradiol (pg/mL) | 36.83 ± 11.41 | 47.18± 26.89 | <0.01 |
| Progesterone (ng/mL) | 0.57 ± 0.23 | 0.85 ± 0.62 | <0.001 |
| FSH (U/L) | 6.73 ± 1.16 | 5.71 ± 1.07 | <0.001 |
| LH (IU/L) | 5.11 ± 1.77 | 10.88 ± 7.57 | <0.001 |
| AFC | 15.35 ± 4.83 | 30.31 ± 11.49 | <0.001 |
| Mean cycle count | 12.86 ± 1.19 | 7.00 ± 2.07 | <0.001 |
| TC | 4.09 ± 0.61 | 4.70 ± 0.77 | <0.001 |
| LDL-C | 2.66 ± 0.49 | 3.26 ± 0.60 | <0.001 |
| HDL-C | 1.41 ± 0.28 | 1.20 ± 0.26 | <0.001 |
| TG | 0.93 ± 0.74 | 1.54 ± 0.81 | <0.001 |
| FBG | 4.98 ± 0.32 | 5.50 ± 0.60 | <0.001 |
| FINS | 6.51 ± 2.43 | 21.40 ± 10.92 | <0.001 |
| HOMA-IR | 1.44 ± 0.55 | 5.31 ± 2.93 | <0.001 |

BMI = body mass index; FSH = follicle-stimulating hormone; LH = luteinizing hormone; AFC = antral follicle count; TC = total cholesterol; LDL-C = low-density lipoprotein cholesterol; HDL-C = high-density lipoprotein cholesterol; TG = triglyceride; FBG = fasting blood glucose; FINS = fasting insulin. Data are presented as mean ± SEM.

**Table S3. Sequences of primers.**

| Gene name | Species | Primer Sequence  (Forward: 5’-3’) | Primer Sequence  (Reverse: 5’-3’) |
| --- | --- | --- | --- |
| *Actin* | Mus musculus | TGTTACCAACTGGGACGACA | GGGGTGTTGAAGGTCTCAAA |
| *Star* | Mus musculus | CTAAACTCACTTGGCTGCTC | TGGTTGGCGAACTCTATCT |
| *Cyp17a1* | Mus musculus | GCCCAAGTCAAAGACACCTAAT | GTACCCAGGCGAAGAGAATAGA |
| *Cyp11a1* | Mus musculus | AGGTCCTTCAATGAGATCCCTT | TCCCTGTAAATGGGGCCATAC |
| *Cyp19a1* | Mus musculus | ACTTCCCTAAGCCCAATG | TCTTCACCTGGAATCGTC |
| *Hsd3β1* | Mus musculus | AGCTCTGGACAAAGTATTCCGA | GCCTCCAATAGGTTCTGGGT |
| *Hsd17β1* | Mus musculus | ACTTGGCTGTTCGCCTAGC | GAGGGCATCCTTGAGTCCTG |
| *Glul* | Mus musculus | TGAACAAAGGCATCAAGCAAATG | CAGTCCAGGGTACGGGTCTT |
| *Gls* | Mus musculus | TTCGCCCTCGGAGATCCTAC | CCAAGCTAGGTAACAGACCCT |
| *Ass1* | Mus musculus | CTCCTGCATCCTCGTGTGG | GCTCACATCCTCAATGAACACC |
| *Nags* | Mus musculus | TACTCCTCCGCAGTCATTACA | CAGCATCCGCTCAGCATTTTT |
